# Thrombospondin 1 in Metabolic Diseases

**DOI:** 10.3389/fendo.2021.638536

**Published:** 2021-03-29

**Authors:** Linda S. Gutierrez, Jovita Gutierrez

**Affiliations:** Department of Biology, Wilkes University, Wilkes Barre, PA, United States

**Keywords:** cardiovascular disease, atherosclerosis, diabetes, angiogenesis, TGFβ1, obesity, nitric oxide

## Abstract

The thrombospondin family comprises of five multifunctional glycoproteins, whose best-studied member is thrombospondin 1 (TSP1). This matricellular protein is a potent antiangiogenic agent that inhibits endothelial migration and proliferation, and induces endothelial apoptosis. Studies have demonstrated a regulatory role of TSP1 in cell migration and in activation of the latent transforming growth factor beta 1 (TGFβ1). These functions of TSP1 translate into its broad modulation of immune processes. Further, imbalances in immune regulation have been increasingly linked to pathological conditions such as obesity and diabetes mellitus. While most studies in the past have focused on the role of TSP1 in cancer and inflammation, recently published data have revealed new insights about the role of TSP1 in physiological and metabolic disorders. Here, we highlight recent findings that associate TSP1 and its receptors to obesity, diabetes, and cardiovascular diseases. TSP1 regulates nitric oxide, activates latent TGFβ1, and interacts with receptors CD36 and CD47, to play an important role in cell metabolism. Thus, TSP1 and its major receptors may be considered a potential therapeutic target for metabolic diseases.

## Introduction

Thrombospondin 1 (TSP1) was discovered in 1971 (1, 2) as a glycoprotein that is secreted by activated platelets, suggesting that the protein’s main function would be associated with hemostasis. However, pioneering studies have uncovered a variety of functions of TSP1, including regulation of cell migration, apoptosis, and angiogenesis (3–6).

TSP1 has a trimeric 450 kDa complex structure that includes a heparin-binding domain with a procollagen homology domain at the amino terminus (7), and type I, II, and III repeats at the carboxyl-terminal end (2, 8). The type I repeats are also called thrombospondin structural repeats (TSR) (9). Within the TSRs, the sequence (CSVTCG) shows a specific affinity for CD36 (also known as fatty acid translocase, FAT) (10). CD36 is a glycosylated protein, member of the class B scavenger receptor family (11). Through binding with CD36, TSP1 induces apoptosis in endothelial cells (4). Importantly, the sequence RFK is located between the first and second TSRs and mediates the activation of the latent form of transforming growth factor beta1(TGFβ1) (12).

**Figure 1:**
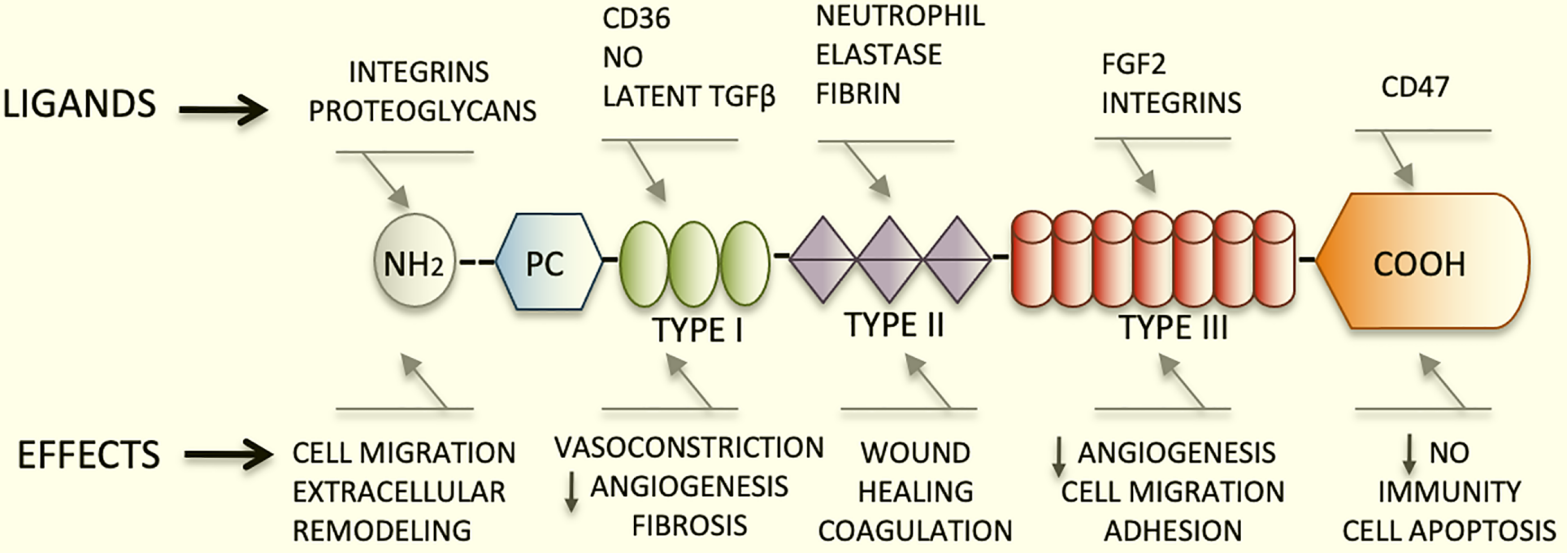
Schematic diagram representing the structure of TSP1, its major ligands and functions. TSP1 displays an amino terminus that interacts with integrins and proteoglycans. The type I, II, and III repeats and the carboxyl-terminal end are also represented herein. The type I repeats also named TSRs contain the binding domain for CD36, responsible for endothelial apoptosis. The sequence RFK that activates the latent form of transforming growth factor beta1(TGFβ1) is also found within these repeats. The type III repeats of TSP1 contain domains that interact with neutrophil elastase and inhibit FGF2. Finally, the carboxyl-terminal domain of TSP1 binds to CD47. This domain interacts with integrins modulating cell adhesion, spreading and migration. TSP1 binds to a diversity of relevant proteins and growth factors not shown in this figure in lieu of clarity.

The type III repeats of TSP1 contain domains that interact with neutrophil elastase (13), and inhibit the angiogenic effects of fibroblast growth factor 2 (FGF2) (14). Finally, the carboxyl-terminal domain of TSP1 shows affinity for CD47, also known as integrin associated protein (IAP), an important TSP1 receptor. CD47 regulates the effects of nitric oxide (NO) in metabolic diseases and has major functions in immunity and hemostasis (15). This domain also interacts with integrins modulating cell adhesion and spreading (**Figure 1**).

TSP1 exerts a wide range of functions as its domains can bind to receptors and specific proteins anchored or secreted in the extracellular matrix. TSP1 is an antiangiogenic protein that modulates cell migration and adhesion; it controls the deposition of collagen in the stroma and modulates immunity (2, 5, 16, 17). Recently, there has been an increasing interest in metabolic alterations as major causes of many pathological conditions. This has rekindled the interest in TSP1 and other matricellular proteins as molecules that may be associated with the modulation of metabolic alterations. This review revisits the functions of TSP1, emphasizes its role in the pathophysiology of major metabolic alterations such as obesity, cardiovascular diseases, and diabetes mellitus, and discusses recent advances in metabolomics that are linked to TSP1.

## TSP1 in Cardiovascular Diseases

TSP1 is weakly expressed in the normal heart (18). In fact, in mice lacking TSP1 (*Thbs1 ^-/-^* mice), minimal effects are observed in heart vascularization, ventricular dimensions, and cardiovascular functions as compared to the control mice (18). However, studies in patients with known cardiovascular conditions have indicated that measuring TSP1 level in plasma is useful for the diagnosis and prognosis of these conditions (19, 20). Moreover, polymorphisms in the *TSP1* gene are linked to a genetic propensity to myocardial infarction, underlining its importance in this condition (21).

In a myocardial infarction model, *Thbs1 ^-/-^* mice display more intense and diffuse inflammation surrounding the infarcted area of the heart (22). These effects of TSP1 seem to be sex-dependent. A preclinical study using a myocardial infarction model showed that female mice displayed lesser heart inflammation than the male mice. Leukocytes were isolated and identified as neutrophils, using antibodies specific for nuclear factor (NF)κB-p65 or peroxisome proliferator-activated receptor (PPAR)γ. The lower concentration of TSP1 in the leukocytes of female mice was evident after 7 days as well as lower levels of IL-6 in plasma (23).

Lesser TSP1 and production of ROS would significantly ameliorate inflammation and reduce the binding of TSP1 with the receptor cluster of differentiation, CD36. This multifunctional receptor has relevant functions in metabolic and immune interactions, including the uptake of circulating fatty acids into the cells (24). CD36 is highly expressed in cardiomyocytes and its deletion reduces lipid uptake and storage, and the induction of genes related to fatty acids metabolism (25, 26). CD36 downregulates 5′-adenosine monophosphate-activated protein kinase (AMPK); however, upon binding with fatty acids, CD36 will promote the activation of AMPK enhancing fatty acid oxidation (27).

TSP1 largely contributes to the rapid remodeling of the extracellular matrix. TSP1 directly and indirectly upregulates and binds to MMPs (28); TSP1 promotes the activation of MMPs by inducing profibrotic genes or by activation of latent TGFβ1. This is an important mechanism in cardiovascular pathology (29). TGFβ1 also accelerates the differentiation of inactive fibroblasts into myofibroblasts and induces the transcription of pro-fibrotic genes. In addition, this growth factor exacerbates the inflammatory response (30). An increase in cleaved TSP1 has been detected in adults with dilated cardiomyopathy, wherein fibrosis is more profuse. These results suggest that TSP1 promotes the activation of latent TGFβ1 and MMPs in the adult cardiac heart, and enhances cardiac muscle remodeling and fibrosis (31), thereby indicating a clear role of TSP1 in cardiovascular pathology during the aging process.

Hypoxia could lead to oxidative stress and production of reactive oxygen species (ROS) by mitochondria. In a model of ischemic reperfusion injury in young and aged hearts, ROS production was induced by the damage with concomitant high expression of TSP1 and dynamin-related protein (Drp-1). Drp-1 is a key protein involved in mitochondrial fission and ROS signaling. Inhibitors of TSP1 may potentially reduce cardiomyocyte damage and aging by reducing production of Drp-1 (32).

Oxidative stress and ROS generation lead to accelerated aging. TSP1, along with CD47, contributes to the aging process in cardiac muscle and endothelial cells. CD47 is expressed in many cell types (33). The binding of CD47 to TSP1 is significant in cardiovascular physiology; notably, the hearts of aging mice deficient in TSP1 and CD47 display elevated heart rates and increased cardiac output. These may be compensatory changes but, levels of cAMP were elevated following a nitric oxide (NO) challenge, suggesting an intrinsic ionotropic and chronotropic activity in the cardiac muscle (34).

The role of TSP1 is quite relevant in vascular diseases such as atherosclerosis. This progressive disease is triggered by endothelial injury and inflammation. Damage to endothelial cells occurs because of changes in blood flow, lipid products, and inflammatory mediators that alter the endothelial barrier. Gradual fibrosis and accumulation of leukocytes and lipids within the intima of the vessel wall occur, and smooth muscle cells (SMCs) migrate from the media in response to cytokines released by inflammatory and immune cells. TSP1 is highly expressed in the arterial wall upon endothelial damage (35) that favors the adherence and penetration of monocytes and macrophages to the arterial wall. TSP1 also enhances the migration and adhesion of macrophages, SMCs, and fibroblasts into the vessel wall and promotes the migration and proliferation of SMCs (36).

During inflammatory conditions such as atherosclerosis, hypoxia inducible factor-1 alpha (HIF1α) enhances the release of interleukin-6, tumor necrosis factor alpha, and TSP1, all of which act as profibrotic factors and alter the vascular homeostasis (8, 37, 38). In pathologies such as pulmonary hypertension, hypoxia inducible factor-2 alpha (HIF2α) is also required for the upregulation of TSP1 in pulmonary vasculature under hypoxic conditions (39).

TSP1 also modulates vasodilation and perfusion of tissues *via* the regulation of NO (34, 40). TSP1 decreases the levels of cAMP and cGMP by inhibiting NO produced by endothelial cells, limiting the smooth muscle cell response, and blocking vasodilation (41–43). Mice lacking TSP1 and CD47 display hypotension, and are more resistant to stress-induced hypertension (15, 44).

TSP1 and CD47 are upregulated in aged arteries (45), and when aortic rings from older individuals were treated with an antibody against CD47, they showed similar sprouting to the aortic rings treated with vascular endothelial growth factor (VEGF). These results revel new and exciting insights into reversing atherosclerosis and the aging process (45).

Lack of TSP1 may contribute to the stabilization of atherosclerotic plaque by inhibiting efferocytosis (46). This function is mediated by the interaction of TSP1 with CD47. CD47 is a surface protein that signals a “Don’t Eat Me” message to phagocytic cells. CD47 inactivates macrophages by binding to the signal regulatory protein alpha (SIRPα) in Mac3^+^ cells (47) and by inhibiting the activation of integrins (48). Similar to TSP1, CD47 is increasingly upregulated during the entire atherogenic process (49). Mice lacking CD47 are more susceptible to atherosclerosis, and treatment with an inhibitory antibody against CD47 induces the same effect as that of decreasing efferocytosis. CD47 deficiency enhances the activation of natural killer cells (NK) by inducing the release of interferon gamma (INFγ). Additionally, treatment of mice with an anti-NK antibody prevents the formation of plaques (47).

**Figure 2:**
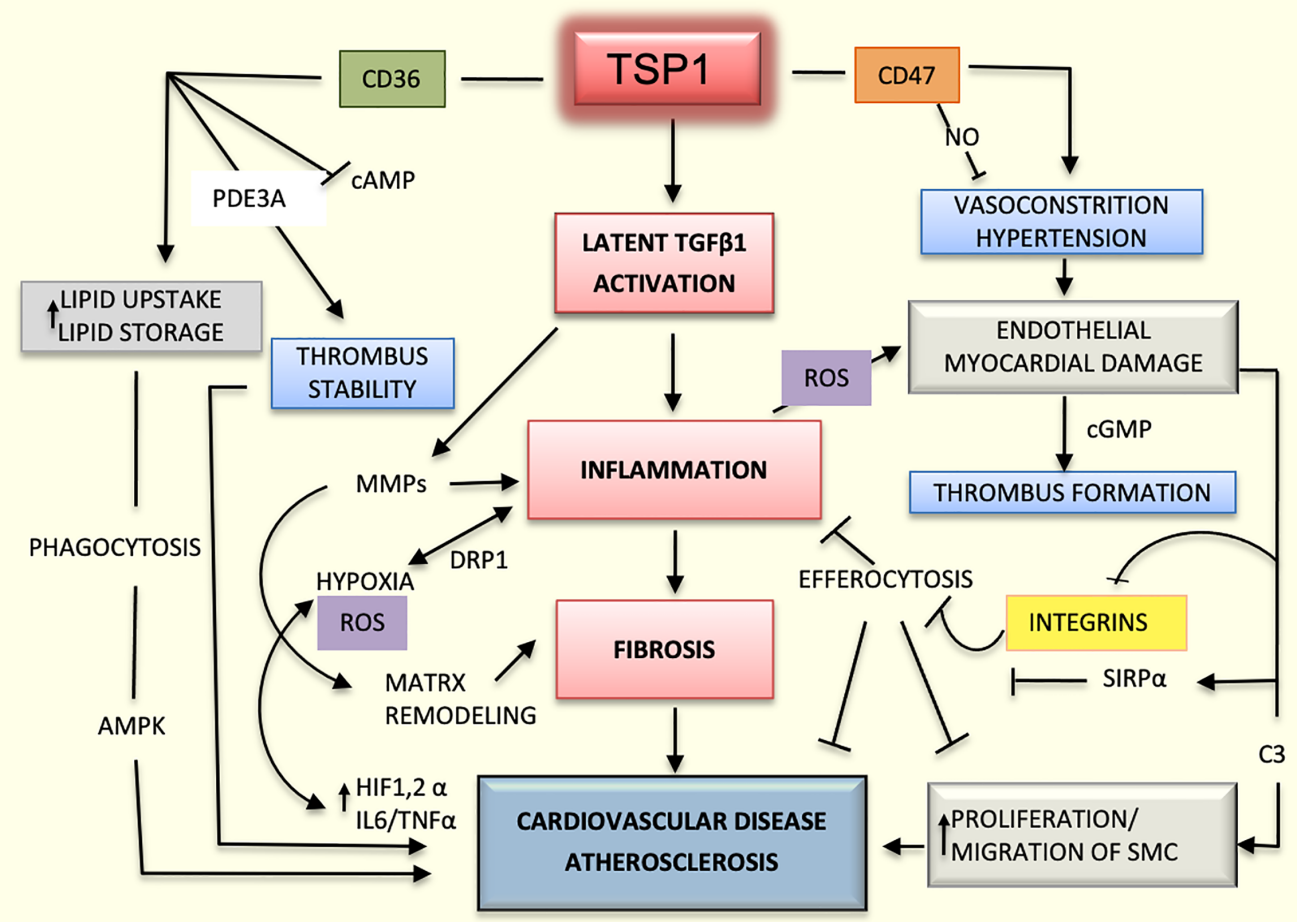
Mechanisms mediated by TSP1 in cardiovascular diseases. The interaction of TSP1 with CD47 inhibits nitric-oxide (NO) levels, thereby decreasing vasodilation and compromising organ perfusion. Hypertension and endothelial injury can in turn, activate the coagulation system, promoting the initiation of thrombus. CD47 suppresses the activation of phagocytic and natural killer (NK) cells during atherogenesis, dampening the inflammatory response and efferocytosis by interacting with signal regulatory protein alpha (SIRPα) and inhibiting integrins. TSP1 activates the latent transforming growth factor beta1 (TGFβ1) enhancing inflammation and fibrosis in the heart and vascular system. Additionally, this growth factor activates matrix metalloproteinases (MMPs), thereby contributing to matrix remodeling in atherosclerosis. Inflammation-induced hypoxia activates hypoxia inducible factor-1 and 2 alpha (HIF1/2α) and increases the levels of interleukin 6 (IL6) and tumor necrosis factor alpha (TNFα) promoting even more the inflammatory process and atherosclerosis. Inflammation also enhances the production of reactive oxygen species (ROS) and cell damage. CD36, as a scavenger receptor B, promotes cellular uptake of lipoproteins and formation of foamy macrophages, which are part of the atherosclerotic plaque. Additionally, by interacting with TSP1, CD36 stabilizes the thrombus in the arterial wall and stimulates the proliferation and migration of smooth muscle cells (SMCs), contributing even further to the atherosclerotic process.

The interaction between TSP1/CD47 is critical for the migration and proliferation of SMCs (50). CD47 can impair the efferocytosis of SMCs thereby, promoting their proliferation (**Figure 2**). Dedifferentiated SMCs from atheromas overexpress CD47 and complement component 3 (C3). C3 is a key protein of the complement system, whose subunits C3a and C3b act as effectors in opsonization, phagocytosis and inflammation. These CD47^+^ and C3^+^ SMCs can evade efferocytosis from polarized M1 murine RAW 264.7 macrophages. Therefore, these SMCs can proliferate and migrate during the process. Additionally, blocking CD47 reestablishes efferocytosis of these SMCs and prevents atherosclerosis (51).

Lack of TSP1 seems to have a protective role in the ApoE^-/-^ mouse model as it is associated with decreased inflammation and improved glucose metabolism (52). However, earlier studies had suggested that TSP1 deficiency promotes atherogenesis and vascular inflammation in the same mouse model (46). It is possible that the functions of TSP1 depend on the vessel type, stage of the lesions, and association with obesity, diabetes, or other metabolic diseases (53). Undoubtedly, the influence of TSP1 on cardiovascular diseases is even more complex and multifactorial due to the variability in activation and functioning of endothelial co-receptors. As an example, TSP1 may activate endothelial receptors CD36 or CD47 differentially or they can induce different responses depending on the type of integrin expressed; for instance, TSP1 inhibits endothelial migration in the microvasculature *via* binding with CD36 (11, 54), but it could also enhance chemotaxis by binding to integrins α3β1and α6β1in HDMEV (7). In addition, possible interactions between CD47 and CD36 should be considered (33). Undoubtedly, the influence of TSP1 on cardiovascular diseases is complex and multifactorial (**Figure 2**).

The activation of latent TGFβ1 *via* TSP1 enhances fibrosis and stiffness in arteries in abnormal conditions without a normal laminar vascular flow (55). These same mechanisms may be implicated in aortic dissection and other arterial pathologies (37, 44, 56). Peptides able to block TSP1 binding to receptors and inhibit its activation of latent TGFβ1may be promising therapeutic agents for cardiovascular diseases and other metabolic diseases (57).

Fibrous plaque leads to ulceration, hemorrhage and scar tissue deposits. Activation of the cascade of coagulation plays an important role in the formation of the atheroma leading to thrombosis and embolism. These complications are first caused by the slowing and turbulence of the blood flow. The next steps involve the adhesion of platelets to the endothelium with the consequent platelet activation and aggregation.

There is evidence of TSP1 as a promoter of platelet aggregation and activation (58). Interestingly, only TSP1 released from alpha granules of activated platelets will suppress cAMP signaling and increase phosphodiesterase 3 (PDE3A) favoring hemostasis (59). CD36 seems to be required, in part, for these processes as platelet-secreted TSP1 can increase the expression of phosphatidylserine by interacting with CD36, enhancing the stabilization of the thrombus (60). Recent studies also show that a CD47-derived peptide, TAX2, inhibits thrombosis by blocking platelet phosphorylation upon contact with collagen. This TAX antibody significantly retarded thrombosis in two models of arterial occlusion (61), and its antithrombotic properties are suspected to be mediated by regulation of NO/cGMP signaling (58). These findings may lead to new therapeutic avenues for metabolic diseases involving thromboembolism.

## TSP1 in Glucose Metabolism and Diabetes Mellitus

In a prediabetic condition, glycemic levels are not high enough to establish a diagnosis of diabetes. However, chronic inflammation, endothelial damage, and extracellular remodeling could lead to diabetes and its complications. TSP1 is associated with all these processes and it is also involved in glucose metabolism as observed in animal and clinical models.

The link between TSP1 and glucose metabolism is evidenced by the fact that aged *Thbs1 ^-/-^* mice show impaired glucose tolerance (62). These mice display hypertrophic pancreatic tissues that produce less proinsulin. Additionally, the pancreas of *Thbs1 ^-/-^* mice release lesser glucose upon insulin secretion (63). This impaired glucose tolerance could be caused by abnormal oxidation of glucose and mitochondrial dysfunction. Further, the pancreatic tissues of *Thbs1 ^-/-^* mice show a higher expression of uncoupling protein 2 (UCP2), which is an inner membrane mitochondrial protein involved in oxidation and ROS metabolism (64). Polymorphisms of UCP2 have been linked to human obesity and diabetes. In addition, islets of *Thbs1 ^-/-^* mice produce more lactate, indicating a switch to glycolysis, which results in reduced production of ATP and proinsulin. This was also confirmed when the islets of *Thbs1 ^-/-^* mice showed higher production of lactate dehydrogenase A, a key glycolytic enzyme responsible for lactate production. These results indicate that TSP1 is vital to maintain the pancreatic homeostasis during normal glycemic conditions.

Vascular perfusion and regulation of angiogenesis are both crucial for the normal functioning of the pancreas and insulin output. The pancreatic islets have more vascularization and blood flow than the acinar component. Additionally, the islet’s endothelium is highly fenestrated and it is actively involved in the production and secretion of insulin. Endothelial cells of the pancreatic islets produce TSP1 (63); however, they also secrete VEGF (62). Reduction of VEGF in islets impairs the release of insulin into the vascular system and these mice with decreased production of VEGF by their pancreatic islets exhibit impaired glucose tolerance as well (65). Therefore, a balance between both proteins is needed to maintain a healthy secretion of insulin and normal glucose blood levels.

Under normal glycemic conditions, the cGMP-dependent protein kinase (PKG) decreases the gene expression of *TSP1* and consequently, the activation of latent TGFβ1 by TSP1. However, during hyperglycemia, TSP1 gene expression is enhanced by the downregulation of PKG (66). This status will lead to the upregulation of the transcription nuclear protein upstream stimulatory factor 2 (USF2). It has been reported that USF2 can bind to a region located in the *TSP1* promoter, and enhance TSP1 transcription (67). Glycosylated proteins such as glycated albumin also modulate TSP1 expression by upregulating USF2 (29). This mechanism could promote higher levels of *TSP1* in diabetes and lead to complications such as diabetic nephropathy. Interestingly, recent studies show that USF2, is in fact, a tumor suppressor that reduces cell proliferation, migration and oxidative stress (68), protective functions also exerted by TSP1 in some inflammatory diseases and cancers (69, 70).

**Figure 3:**
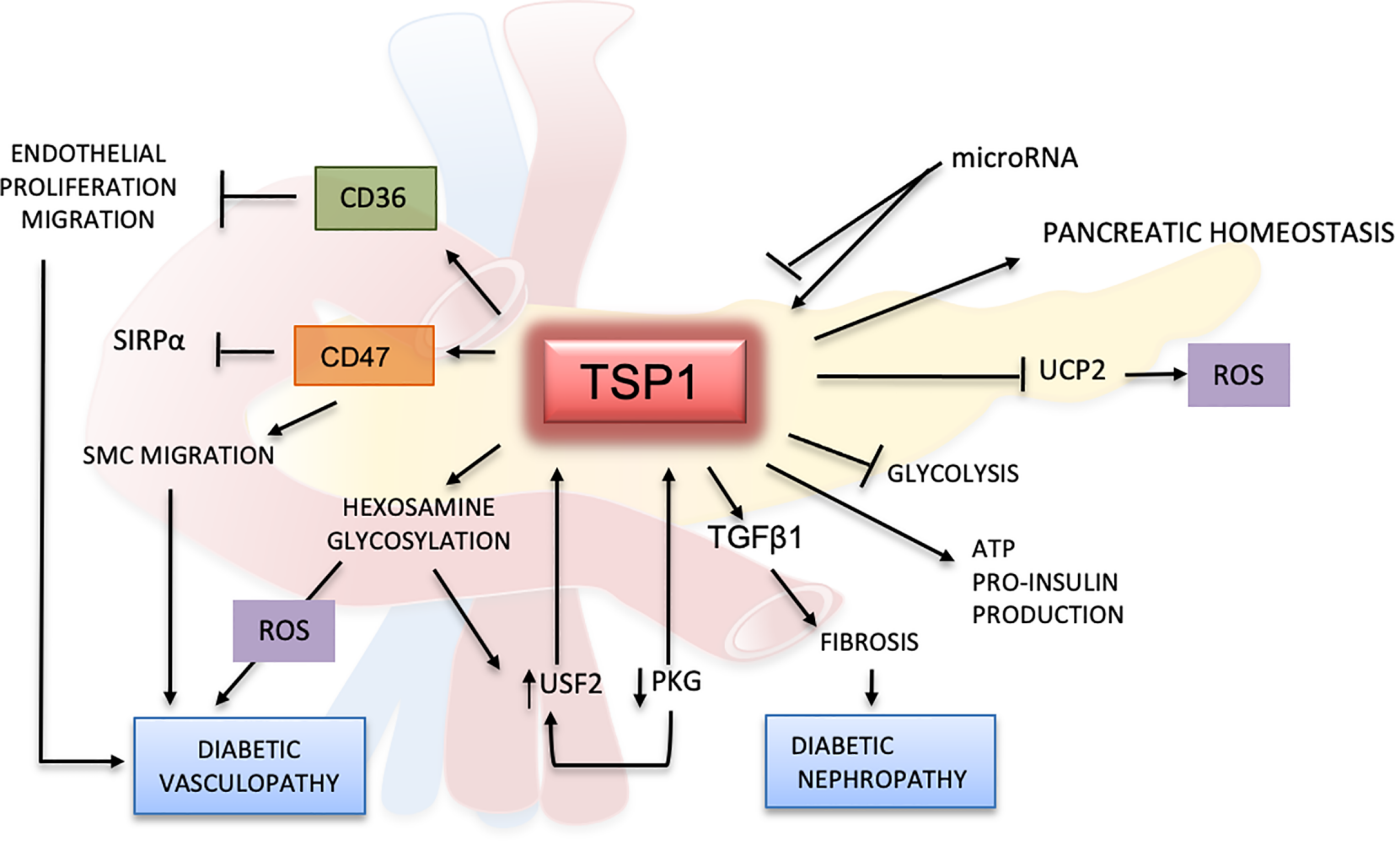
Effects of TSP1 and ligands in glucose metabolism and diabetes mellitus. TSP1 protects the pancreatic endothelium and controls angiogenesis by enhancing insulin production and release. TSP1 also blocks the formation of uncoupling protein 2 (UCP2), a mitochondrial protein involved in oxidation and reactive oxygen species (ROS) metabolism. Under normal glycemic conditions, TSP1 inhibits glycolysis promoting the production of more ATP and pro-insulin. However, under hyperglycemic conditions and by activating TGFβ1, TSP1 accelerates the inflammation and fibrotic changes in multiple organs, especially in the kidney, leading to diabetic nephropathy and other diabetic complications. The TSP1 receptors CD36 and CD47 can inhibit the proliferation and migration of endothelial and smooth muscle cells, thereby contributing to endothelial dysfunction in diabetes. In this context, CD47 regulates SMC migration by binding mainly to TSP1 while limiting its association with SIRP1α. Activation of the hexosamine pathway and glycosylated products can induce further endothelial damage, with generation of ROS and delay of the wound-healing process in diabetes. Hyperglycemia will inhibit PKG and induce upstream stimulatory factor 2 (USF2), promoting the upregulation of TSP1 in diabetes. However, all these effects mediated by TSP1 in the glucose metabolism may be regulated by microRNAs.

Undoubtedly, high levels of glucose promote oxidative stress (71, 72). Oxidative stress, insulin resistance, and increased levels of glycosylated products likely lead to endothelial dysfunction in hyperglycemia (73). Glycosylation and the expression of TSP1 in SMCs could be mediated by activation of the hexosamine pathway (**Figure 3**). Sugars and activators of this pathway may directly enhance the proliferation of SMCs and promote the transcriptional expression of TSP1. The treatment of SMCs with an anti-TSP1 antibody and a TSP1 siRNA reverses their proliferation, thereby suggesting a direct link between the hexosamine catabolic pathway, glycosylation, and TSP1 in diabetes (36, 67).

High expression of TSP1 has been observed in SMCs during hyperglycemia. CD47 regulates the migration of SMCs under hyperglycemic conditions by interacting with SIRPα (50). This binding blocks the migration of SMC mediated by the insulin-like growth factor 1 receptor (IGF1R) signaling (50). When CD47 binds to TSP1, or to a peptide derived from the CD47 domain of TSP1, the binding of CD47 to SIRPα is reduced due activation of the IGF1R signaling (50). However, high levels of glucose protect CD47 from degradation and promote its association with SIRPα (74, 75). Further, protein and mRNA levels of TSP1 and its receptor CD47 were enhanced in endothelial cells isolated from wound lesions of diabetic Wistar rats (76). By using a CD47-blocking siRNA, endothelial cells show increased proliferation, migration, and tube formation. These results suggest that angiogenesis in diabetes may be regulated by the TSP1-CD47 axis (76). As age predisposes the body to insulin resistance and hyperglycemia, CD47 will further inhibit SMCs proliferation and angiogenesis. Indeed, CD47-null aging mice have demonstrated better responses to glucose than control mice (45).

Currently, novel antibodies inhibiting the interaction between SIRPα and CD47 are in preclinical phases, while other anti-CD47 antibodies such as AO-176 are currently under evaluation in clinical trials for the treatments of several types of cancers. Some of these antibodies target tumor macrophages, enhancing efferocytosis of tumor cells (77, 78).

CD36 also plays a significant role in lipid and glucose metabolism, and can promote insulin resistance and hyperinsulinemia (79, 80). However, its functions in these conditions seem contradictory. In a clinical trial that involved patients with a metabolic syndrome, CD36 mRNA was downregulated in the blood mononuclear cells of patients who consumed a healthy low-fat diet (81). In another study, decreased expression of CD36 in muscle was associated with increased risk of type 2 diabetes (82). In fact, deficiency of human CD36 is frequently observed in Asian and African populations, and is linked to insulin resistance (80). In spite of this, mice lacking CD36 are protected from insulin resistance, even under a high-fat diet (25).

CD36 is a multifunctional protein with effects that could be independent of its interaction with TSP1. Instead of having a concerted effect, TSP1 could block the translocase fatty acid activity of CD36 in metabolic syndrome (83). Conversely, the addition of TSP1 or the mimetic peptide ABT526 to hepatocytes cultured under high sucrose levels and insulin significantly decreased hepatic steatosis. In this study, TSP1 inhibited sterol regulatory element-binding protein 1 (SREBP1), an insulin-activated protein with important functions in glucose and lipid metabolism. SREBP1 regulation mediated by TSP1 seems to require CD36 as these results were not reproduced in CD36-deficient hepatocytes (84).

CD36 may have different effects depending on the type of cell and organ in which it is expressed; for example, loss of CD36 reduces the expression of genes related to glucose metabolism in cardiomyocytes but enhances their expression in endothelial cells (85). Additionally, mice lacking CD36, when fed with a low fat diet, show high gluconeogenesis and decreased hepatic glycogen levels; indicating a prediabetic status that seems to be conditional to the type of diet consumed (86). Further research is needed to understand the specific role of CD36 and its interactions with TSP1 in diabetes.

Adding more complexity to the role of TSP1 in hyperglycemia is the fact that its expression could be regulated by microRNAs. Overexpression of miR-467 inhibits TSP1 secretion by endothelial cells during hyperglycemia, and enhances angiogenesis in breast tumors (87). In addition, microRNAs can upregulate TSP1 and genes involved in the TGFβ1 pathway (88). Furthermore, microRNAs can protect pancreatic cells from damage induced by free fatty acids (FFAs). This was demonstrated when a rat insulinoma cell line (INS) was transfected with a TSP1 vector, and the transfected cells became considerably resistant to palmitate (a FFA)-induced apoptosis (89). Conversely, when INS cells were transfected with the 3′-UTR of TSP1 and miR-182-5p, they showed a significant decrease in TSP1 expression, indicating that this microRNA was able to target TSP1 (89).

Diabetes mellitus leads to severe complications involving the cardiovascular system, kidney, retina, and nervous system, resulting in a persistent microvascular injury in tissues and delayed healing (90). The slow wound healing observed in diabetes, could be explained in part by the elevation of TSP1 and inhibition of VEGF observed during capillary regression under hyperglycemic conditions (91). In addition, through its binding to CD36 or even CD47, TSP1 could prevent tube formation and migration of endothelial cells and depress the phagocytic response, hindering the healing process. TSP1 is also involved in the pathophysiology of diabetic cardiomyopathy, obesity, and neuropathy (92–95). Indeed, neuropathy is one of the most devastating sequelae of diabetes, and nervous system damage has been associated with impaired angiogenesis and increased TSP1 expression (96).

One common complication of diabetes is nephropathy. Both TSP1 and TGFβ1 are involved in this condition. TSP1 is highly expressed in glomerular mesangial cells as diabetic nephropathy progresses (97). Activation of latent TGFβ1, mediated by TSP1, is necessary for the normal functioning of pancreatic islets. However during chronic hyperglycemia, TGFβ1 can worsen diabetic nephropathy by inducing fibrosis (98). Thus, blocking this activation could be an alternative mechanism for preventing this complication. For instance, fibrosis in rat peritoneal tissues can be reversed by the treatment with a TSP1-derived peptide. This peptide blocks the activation of latent TGFβ1, and significantly reduces fibrosis both *in vivo* and *in vitro* (99).

Clinical studies suggest that TSP1 could be a potential diagnostic marker for diabetes. In patients with glucose intolerance, TSP1 mRNA levels in adipose tissues were significantly lower when patients were treated with pioglitazone, a drug that improves the response to insulin (94). In a randomized study, plasma samples from 398 patients with prediabetes were evaluated, and proteomics studies indicated that TSP1 was correlated with high levels of glucose after 2 h of fasting (100). Reduced levels of TSP1 in serum positively correlated with improved glucose intolerance and diminished liver fat content (84). In another clinical trial evaluating metabolic syndrome, TSP1 serum levels showed a strong association with high HbA1c in males (101). These results confirm TSP1 as a protein linked to hyperglycemic mechanisms but further research is required to better understand its role in glucose metabolism and diabetes (**Figure 3**).

## TSP1 in Adipocyte Metabolism and Obesity

Adipose tissue is one of the most highly vascularized tissues of the body, and angiogenesis is expected to be involved in its remodeling and metabolism. Besides the well known role of TSP1 in the inhibition of angiogenesis (6, 102), early studies have shown that a lack of TSP1 could decrease abdominal fat without resulting in any change to vascular density (103). *Thbs1 ^-/-^* mice are resistant to obesity following ingestion of a high-fat diet and display normal levels of leptin, an important adipokine associated with increased body weight (104). However, studies have demonstrated that TSP1 is upregulated in the visceral fat of obese mice; similar findings were reported in clinical studies as well (104–107).

Adipogenesis as well as the growth and distribution of the adipose tissues are associated with angiogenesis. Endothelial cells produce cytokines and growth factors such as VEGF that fuel lipogenesis and adipose tissue expansion (108). These pro-angiogenic factors could also promote the trans differentiation of white adipocytes into brown adipocytes. However, a decrease in vascularity has been observed through the transition of brown fat into white adipose tissue during obesity (109, 110). TSP1 may be involved in these processes by interacting with Argonaute 1(AGO1) a protein required for gene silencing (111). Downregulation of TSP1 was detected in endothelial cells and adipose tissues of mice lacking AGO1 fed with a high fat, high sugar diet. These mice showed lower body weight and normal insulin response (112). Their adipose tissues display higher vascularity and typical features of brown fat compared to control mice. Conversely, TSP1 and AGO1 were upregulated in endothelial cells from obese and diabetic type 2 donors, suggesting that TSP1 may promote obesity and insulin resistance by reducing angiogenesis.

Certainly, TSP1 contributes to adipose tissue metabolism in several ways apart from performing its antiangiogenic functions. The relevance of TSP1 in inflammation and obesity seems to be mediated principally by macrophages. The phenotype of *Thbs1^-/-^* macrophages is characterized by lower pro-inflammatory activation and by decreased secretion of cytokines (113, 114). Adipose tissues that lack TSP1 display reduced leukocytic infiltration, and *Thbs1 ^-/-^* macrophages secrete lower levels of inflammatory cytokines (115). In a diet-induced animal model of obesity, the influx of CD68^+^ macrophages and angiogenesis in *Thbs1 ^-/-^* adipose tissues was similar to that of the control tissues (113). Moreover, sensitivity to insulin was also similar to that observed in the *Thbs1 ^+/+^* obese mice. However, obese mice with a targeted deletion of TSP1 only in F4/80^+^ macrophages show impaired leukocytic infiltration in the adipose tissues. Additionally, these adipose tissues display reduced fibrosis, improved insulin resistance, and decreased production of pro-inflammatory cytokines (52). These changes were more evident when obesity was already established, indicating a chronic and progressive mechanism.

These same studies showed a decrease in the activation of latent TGFβ1, both *in vitro and in vivo*, and indicated that TSP1 deficiency and a decline in latent TGFβ1 activation could be protective against inflammation and obesity. Peptides derived from the 3TSR of TSP1 containing the domain able to activate the latent TGFβ1 have been shown to enhance the influx of CD68+ macrophages in models of inflammation and cancer (116, 117).

As obesity is considered a chronic inflammatory condition, signaling between macrophages and adipocytes occurs during its development and progression. CD36 significantly contributes to enhance the macrophages’ inflammatory functions in adipose tissues (118). The interaction of CD36 with TSP1 may also regulate the damage induced by saturated FFAs during obesity and dyslipidemia. The high expression of TSP1 after the treatment of podocytes with FFA corroborates these results. Additionally, FFA-induced podocyte apoptosis was inhibited in *Thbs1 ^-/-^* and *CD36 ^-/-^* podocytes, and they were induced when they were treated with a CD36 peptide (119).

Recent evidence indicates that CD36 signaling is dependent on the activation of STAT3 in tumor cells and adipose tissue (120). High fat diet increases phosphorylation of the signal transducer and activator of transcription 3 (STAT3) *via* activation of CD36. STAT3 is an important transcription factor in inflammation and immunity. It is also well known that inflammation is a landmark of adipose tissues in obesity. A relationship between inflammation and STAT3 was reported in *Thbs1 ^-/-^* mice in a model of colitis. These colonic tissues show more inflammation and enhanced phosphorylation of STAT3 as well. A mimetic peptide containing the TSP1 binding domain for CD36 reverted the STAT3 activation (121).

*CD47 ^-/-^* mice, as well as *Thbs1 ^-/-^* mice, are resistant to obesity (52, 104). The subcutaneous fat tissue of *CD47 ^-/-^* mice displays lower expression of TSP1, but higher expression of cGMP (122) which is integral in cardiovascular and adipose metabolism. This signaling regulates the proliferation, differentiation and secretory activity of brown adipocyte depots (123, 124). Moreover, mitochondria from *CD47 ^-/-^* brown adipocytes display anomalies in shape and high metabolic activity. These results suggest that CD47 may be a potential target for reducing obesity, as increased body distribution and activity of brown fat in adults are regarded as a potential strategy for reducing obesity.

**Figure 4:**
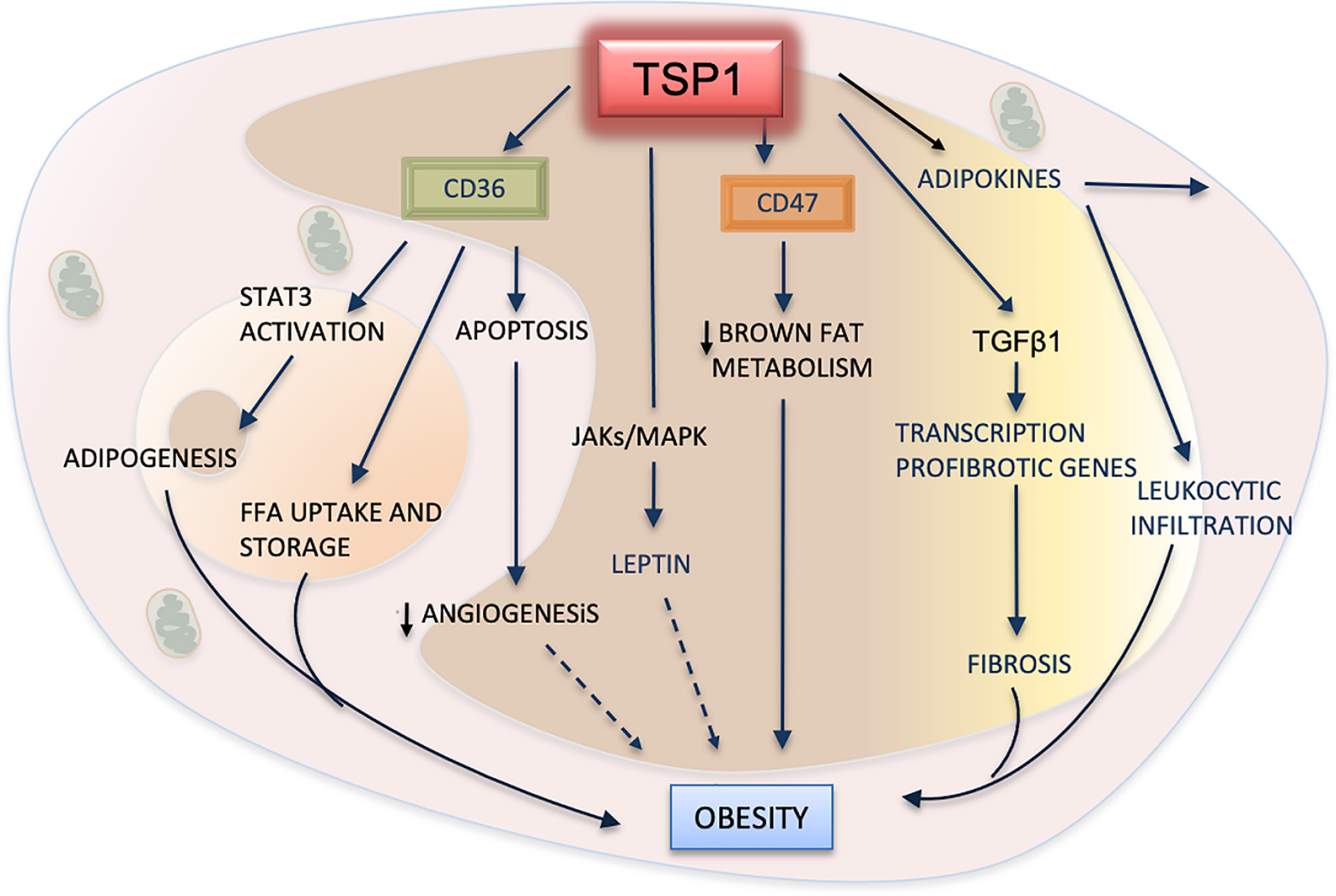
TSP1 in adipose metabolism and obesity. TSP1 activates the latent form of transforming growth factor beta1 (TGFβ1), consequently promoting the recruitment of leukocytes, the influx of macrophages to the adipose tissue, and the release of inflammatory cytokines and adipokines. TGFβ1 will also contribute to fibrosis of adipose tissues by inducing the transcription of pro-fibrotic genes. By interacting with TSP1, CD47 suppresses the mitochondrial metabolism in brown adipose cells, therefore exhibiting a pro-obesity effect. TSP1 interacts with the hormone leptin by activating the JAK/STATs pathway. Increased plasma levels of leptin are correlated with high body fatness. The dashed arrow indicates a probable but yet unexplained association with obesity. CD36 facilitates the uptake of free fatty acids (FFA) into adipose cells, but it can also promote the apoptosis of endothelial cells, decreasing angiogenesis. Perhaps, this could contribute to the lower vascularization observed in white adipose tissues during the progression of this condition (dashed arrow). Finally, CD36 can regulate adipogenesis and expansion of adipose cells by activating the signal transducer and activator of transcription 3 (STAT3) signaling.

Adipose tissue is an endocrine organ that produces several hormones, including the adipokine leptin, a peptide secreted by adipocytes (125). Leptin receptors are involved in appetite, food intake and insulin secretion. Though, its involvement in obesity is still uncertain (**Figure 4**), several animal and clinical studies have demonstrated that increased plasma levels of leptin are associated with high body mass and atherosclerosis (126). However, recent studies have shown that *ApoE* ^-/-^/*Thbs1 ^-/-^* mice treated with exogenous leptin display significant decreases in body weight and attenuation of atherosclerosis. Levels of VLDL and triglycerides were substantially lower in these double null mice even after being fed with a western high-fat diet. These effects, mediated by TSP1 *via* leptin, seem to be induced by the upregulation of JAK2 and MAPKs signaling pathways (127). Activation of these pathways has been also implicated in the upregulation of TSP1 by leptin in vascular muscle cells during atherogenesis (128).

TSP1 could be a marker for weight loss and higher basal metabolism. In a clinical study, high TSP1 expression was detected in the visceral fat of patients undergoing a weight loss program consisting of low caloric intake and increased physical activity (101). TSP1 serum levels of these patients were higher, and correlated with abdominal obesity, hypertension, hyperglycemia and high levels of leptin. These associations were observed particularly in premenopausal females. However, these results should be interpreted cautiously as TSP1 levels in serum may result from platelet activation, and may not reflect the high physiological concentration of TSP1 observed in plasma samples. Platelet function can be inhibited in obese patients as well (129). Therefore, low levels of TSP1 could be caused by impairment of platelet activation, which may be enhanced by weight loss. However, the same results were observed in another clinical study when proteomic analyses were performed in peripheral blood mononuclear cells and visceral adipose tissues. TSP1 was again one of the proteins upregulated in obese patients, and TSP1 levels were decreased after the same patients followed an exercise regimen (130). As obesity and its complications are a significant health concern in our society, the premise that TSP1 could be a diagnostic marker for obesity compellingly merits further investigation.

## Conclusions and Future Perspectives

TSP1 is a frequently studied and well-characterized matricellular protein. Its antiangiogenic effect in cancer was previously explored in animal studies and clinical trials. As TSP1 contains multiple binding sites to several receptors and growth factors, a more sophisticated understanding of its dynamic functions is necessary. As we have learned more about the intrinsic mechanisms involving TSP1 and its interactions, it has become clear that these mechanisms may be tissue-specific and depend on the affinity and temporal/spatial expression of these cofactors within the tissue environment.

In the past, pioneers in the field developed a foundation for understanding the pathophysiological roles of TSP1 in metabolic diseases. As new scientific tools and more in-depth metabolomics approaches are now possible, new mechanistic insights have emerged about TSP1. Among these exciting breakthroughs is the development of novel antibodies blocking TSP1/CD47 interactions, some of which have progressed to clinical trials for treating several types of cancers. One such antibody, TAX, has shown promising results as an antithrombotic, leading to its potential therapeutic use in cardiovascular diseases and other metabolic conditions.

The potential use of TSP1 as a diagnostic and prognostic indicator of diabetes and obesity is an exciting possibility, as well as the use of microRNAs to mitigate the effects of TSP1 in these diseases. More insights regarding the role of this protein in mitochondrial metabolism and immunity are emerging. These scientific advances position TSP1 and its receptors as important therapeutic targets in heart disease, diabetes, obesity, aging and cancer.

## Author Contributions

LG conceived and designed the study, collected and analyzed the data, and wrote the manuscript. JG collected and analyzed the data, contributed to figure creation, and reviewed the manuscript. All authors contributed to the article and approved the submitted version.

## Funding

Financial support for this work was provided by the Provost Research and Scholarship Funds at Wilkes University.

## Conflict of Interest

The authors declare that the research was conducted in the absence of any commercial or financial relationships that could be construed as potential conflict of interest.

## Abbreviations

TSP1, thrombospondin 1; TGFβ1, transforming growth factor beta 1; *Thbs1 ^-/-^* mice, mice lacking TSP1 protein; CD47, cluster of differentiation 47; CD36, cluster of differentiation 36; MMP, matrix metallopeptidase; AMPK, 5′-adenosine monophosphate-activated protein kinase; NO, nitric oxide; cAMP, cyclic adenosine monophosphate; HIF1α, hypoxia inducible factor 1 alpha; IL-6, interleukin- 6; TNFα, tumor necrosis factor alpha; ApoE, apolipoprotein E; VEGF, vascular endothelial growth factor; siRNA, small interfering RNA; Mdm2, mouse double minute 2 homolog; let-7d, lethal-7d; ROS, reactive oxygen species; USF2, upstream stimulatory factor 2.
